# Global ecological success of *Thalassoma* fishes in extreme coral reef habitats

**DOI:** 10.1002/ece3.2624

**Published:** 2016-12-20

**Authors:** Christopher J. Fulton, Peter C. Wainwright, Andrew S. Hoey, David R. Bellwood

**Affiliations:** ^1^ Research School of BiologyThe Australian National UniversityCanberraACTAustralia; ^2^ Department of Evolution & EcologyUniversity of CaliforniaDavisCAUSA; ^3^ ARC Centre of Excellence for Coral Reef StudiesCollege of Marine & Environmental SciencesJames Cook UniversityTownsvilleQLDAustralia; ^4^ Red Sea Research CenterDivision of Biological and Environmental Science and EngineeringKing Abdullah University of Science and TechnologyThuwalSaudi Arabia

**Keywords:** aspect ratio, ecomorphology, labriform, macroecology, specialization

## Abstract

Phenotypic adaptations can allow organisms to relax abiotic selection and facilitate their ecological success in challenging habitats, yet we have relatively little data for the prevalence of this phenomenon at macroecological scales. Using data on the relative abundance of coral reef wrasses and parrotfishes (f. Labridae) spread across three ocean basins and the Red Sea, we reveal the consistent global dominance of extreme wave‐swept habitats by fishes in the genus *Thalassoma*, with abundances up to 15 times higher than any other labrid. A key locomotor modification—a winged pectoral fin that facilitates efficient underwater flight in high‐flow environments—is likely to have underpinned this global success, as numerical dominance by *Thalassoma* was contingent upon the presence of high‐intensity wave energy. The ecological success of the most abundant species also varied with species richness and the presence of congeneric competitors. While several fish taxa have independently evolved winged pectoral fins, *Thalassoma* appears to have combined efficient high‐speed swimming (to relax abiotic selection) with trophic versatility (to maximize exploitation of rich resources) to exploit and dominate extreme coral reef habitats around the world.

## 1 Introduction

Abiotic stress can be a dominant selective force that constrains the demography and ecological niche space of organisms (Hutchinson, 1957). Phenotypic innovations, however, can allow organisms to create ecological opportunities that relax abiotic selection and facilitate ecological releases in the form of increased population sizes, broader resource use, and/or increased diversification (Hunter, 1998; Yoder et al., 2010). Indeed, some of the most dramatic ecological releases of animals from aquatic to terrestrial and aerial habitats are thought to have arisen from key morphological innovations such as jointed limbs (Daeschler, Shubin, & Jenkins, 2006) and feathers (Prum, 1999). More subtle morphological changes can be just as important, such as relatively small changes in the limb length of *Anolis* lizards that increase climbing performance and facilitate adaptation to arboreal habitats (Losos, Warhelt, & Schoener, 1997; Yoder et al., 2010). In many cases, locomotor traits have played a key role in helping animals overcome environmental challenges to occupying vastly different habitats.

In fishes, a range of locomotor traits have been linked to extremes in habitat occupation. Some have involved exaptation of existing aquatic body plans, such as the unique axial movements, oral suction, and other behaviors used by amphibious fishes to move across the aquatic–terrestrial interface (e.g., Hsieh, 2010; Schoenfuss & Blob, 2003). Development of more specialized morphological structures has also helped fish gain access to extreme habitats, such as the pelvic suction disk that allows stream gobies to occupy high‐flow rapids and waterfalls (e.g., Donaldson, Ebner, & Fulton, 2013; Schoenfuss & Blob, 2003). In most cases, these traits have driven enhancements in locomotor performance (speed, agility, and/or efficiency) to gain access into extreme habitats. The extent of ecological success (i.e., relative abundance of species occupying similar niches) that has arisen from these locomotor adaptations, however, remains largely unexplored for fishes, particularly at macroecological scales.

Wave energy is a ubiquitous abiotic stress acting upon aquatic biota in shallow waters. In shallow coral reef habitats exposed to incident wave energy, intense water motion can be challenging for fish locomotion (Fulton, 2010), stability (Heatwole & Fulton, 2013), and foraging (Noble, Pratchett, Coker, Cvitanovic, & Fulton, 2014). However, gaining access to such wave‐swept habitats could confer key benefits, such as an abundance of trophic resources (e.g., detritus and algal turfs, Crossman, Choat, Clements, Hardy, & McConochie, 2001; Russ, 2003; benthic invertebrates, Kramer, Bellwood, & Bellwood, 2014). Some coral reef fishes possess winged pectoral fins (indicated by a high aspect ratio; Wainwright, Bellwood, & Westneat, 2002) that confer high swimming speed performance and extreme energetic efficiency (Fulton, Bellwood, & Wainwright, 2005; Fulton, Johansen, & Steffensen, 2013a). While such capabilities seem well suited to living in habitats characterized by high wave‐driven flows (Fulton, 2010), it remains to be established whether this phenotypic adaptation has conferred broad ecological success for fish taxa that occupy such extreme habitats.

We investigated the relative abundance of labrid species occupying wave‐swept coral reef habitats to determine whether fishes that possess a key locomotor adaptation have achieved global ecological success via numerical dominance over their sympatric confamiliars. We examine whether the ecological success of taxa varies across a sixfold global gradient in the species richness of confamiliars (a proxy for competitive pressure), and whether it is dependent on the intensity of wave energy impinging on shallow reef habitats. In identifying the dominant taxa among locations and wave exposures, we explored the macroecological consequences arising from an ecologically relevant adaptation (winged pectoral fins) that has independently evolved among several lineages of ray‐finned fishes (Fulton, 2010; Fulton et al., 2005). We focused upon fishes of the family Labridae, because they are one of the most diverse and abundant groups of coral reef fishes found across the globe (Bellwood & Hughes, 2001).

## 2 Materials and Methods

Global variations in the relative abundance of labrid fishes occupying shallow (3 m depth) wave‐swept crest habitats were investigated for eleven coral reef locations across three ocean basins and the Red Sea (Table 1). We used underwater visual surveys to record the total number of wrasses and parrotfishes encountered within 20‐min timed‐swim belt transects, following the exact method of Bellwood and Wainwright (2001). Four transects were swum within the crest habitat on each reef, at two reefs per location (except in Panama, where just one set of four transects could be taken in the Las Perlas Archipelago). Species were ranked by density (individuals 100 m^−2^), and the total number of reef crest labrid species (richness) tallied for each location. To explore the relationship between species richness and relative abundance, we divided the density of the most abundant species by the density of the second most abundant species as a conservative measure of numerical dominance. We then explored the extent to which numerical dominance of the most abundant species varied across a sixfold gradient in labrid species richness, which can represent a gradient of competitive pressure from confamiliar fishes that tend to have very similar resource requirements (Cahill, Kembel, Lamb, & Keddy, 2008; Darwin, 1859; Webb, Ackerly, McPeek, & Donoghue, 2002).

**Table 1:** List of global survey locations, total labrid species richness recorded within the crest habitat, mean density (fish 100 m^−2^ ± standard error), and pectoral fin aspect ratio (AR) of the top three species per location. n/a, not available

Location	Species richness	Three most abundant species	Density ± *SE*	Pectoral AR
Panama	10	*Thalassoma lucasanum*	75.3 ± 38.5	1.81
		*Halichoeres dispilus*	11.1 ± 4.6	1.50
		*Halichoeres chierchiae*	3.8 ± 2.0	n/a
Caribbean	18	*Thalassoma bifasciatum*	103.3 ± 20.9	1.86
		*Halichoeres bivitattus*	13.5 ± 2.8	1.37
		*Halichoeres maculipinna*	10.5 ± 1.5	1.39
Red Sea	26	*Thalassoma rueppellii*	89.3 ± 6.0	1.90
		*Gomphosus caeruleus*	6.1 ± 1.0	1.65
		*Stethojulis albovittata*	1.5 ± 0.8	1.86
Micronesia	32	*Thalassoma quinquevittatum*	45.2 ± 12.2	1.97
		*Thalassoma amblycephalum*	26.8 ± 5.6	1.84
		*Halichoeres margaritaceus*	13.7 ± 4.5	1.38
French Polynesia	34	*T. quinquevittatum*	63.0 ± 15.5	1.97
		*Thalassoma hardwicke*	11.6 ± 3.3	1.90
		*Stethojulis bandanensis*	7.6 ± 1.9	2.08
Samoa	35	*T. quinquevittatum*	28.8 ± 6.0	1.97
		*T. hardwicke*	4.1 ± 1.2	1.90
		*H. margaritaceus*	4.1 ± 1.1	1.38
Mauritius	38	*Thalassoma genivittatum*	25.1 ± 3.4	1.95
		*T. hardwicke*	7.1 ± 2.3	1.90
		*S. albovittata*	6.0 ± 1.3	1.86
Great Barrier Reef	45	*T. amblycephalum*	106.4 ± 19.1	1.84
		*Thalassoma jansenii*	21.9 ± 6.0	1.96
		*T. quinquevittatum*	20.2 ± 2.7	1.97
Cocos/Rowley Shoals	51	*T. quinquevittatum*	35.0 ± 5.0	1.97
		*T. amblycephalum*	21.2 ± 5.6	1.84
		*Halichoeres marginatus*	8.4 ± 1.7	1.54
Indonesia	56	*T. amblycephalum*	30.0 ± 11.2	1.84
		*T. hardwicke*	16.9 ± 4.0	1.90
		*Pseudocheilinus hexataenia*	8.0 ± 2.8	0.91
Papua New Guinea	61	*T. amblycephalum*	54.8 ± 20.8	1.84
		*T. hardwicke*	15.7 ± 2.4	1.90
		*Halichoeres miniatus*	10.4 ± 7.5	1.29

To examine how incident wave energy influenced the relative abundance of *Thalassoma* (the most abundant labrid genus) and all other genera, we quantified the abundance of all labrid fishes within shallow reef habitats (3 m depth) that are either sheltered (back reef) or directly exposed (crest) to incident trade wind‐driven waves across a 40‐km cline on the northern Great Barrier Reef (GBR), Australia (Fulton, Binning, Wainwright, & Bellwood, 2013b). Our GBR surveys comprised four replicate belt transects (timed swims as above) taken within each habitat zone (back, crest) of two reefs at each of three continental shelf positions (inner‐, mid‐ and outer‐shelf). Corresponding measures of maximum in situ habitat flow velocities were taken at the same GBR reefs, using three dynamometers (Bell & Denny, 1994) deployed simultaneously at 5 m depth within the reef back and crest habitats of the same six reefs, during an 8‐day period when south‐easterly winds gusted above 20 knots daily (Fulton et al., 2013b). We used a permutational multivariate analysis of variance (Anderson, Gorley, & Clarke, 2008) on a binomial deviance dissimilarity matrix (Anderson & Millar, 2004) constructed from the log_10_(*x* + 1)‐transformed data to explore differences in the relative density of *Thalassoma* versus other genera with the fixed factors of continental shelf position and habitat zone (crest/back), and random reef replicates nested within shelf position. A minimum of 9,500 permutations of residuals under a reduced model were performed using type III sums of squares within the PERMANOVA+ (v1.0.6) module of Primer (v6.1.16). We then fitted least‐squares linear and nonlinear (quadratic) regressions between fish density and mean habitat flow velocities to explore trends in the abundance of the two fish groups. All regression were fitted using Sigmaplot (v9.01). To examine links with fin morphology, we plotted mean density against the pectoral fin aspect ratio for each species present within the most exposed reef crests of the outer Great Barrier Reef, and documented the pectoral fin aspect ratio for the top three species present in each of our global locations. Pectoral fin aspect ratios were mostly taken from published sources (Fulton et al., 2005; Wainwright et al., 2002). New values for six species (*Halichoeres dispilus*,* H. chierchiae*,* Stethojulis albovittata*,* T. genivittatum*,* T. rueppellii,* and *T. lucasanum*) were calculated from calibrated images taken by the first author or by J. E. Randall (available on Fishbase, Froese & Pauly, 2016), following the exact method of Fulton et al. (2005).

## 3 Results

**Figure 1:**
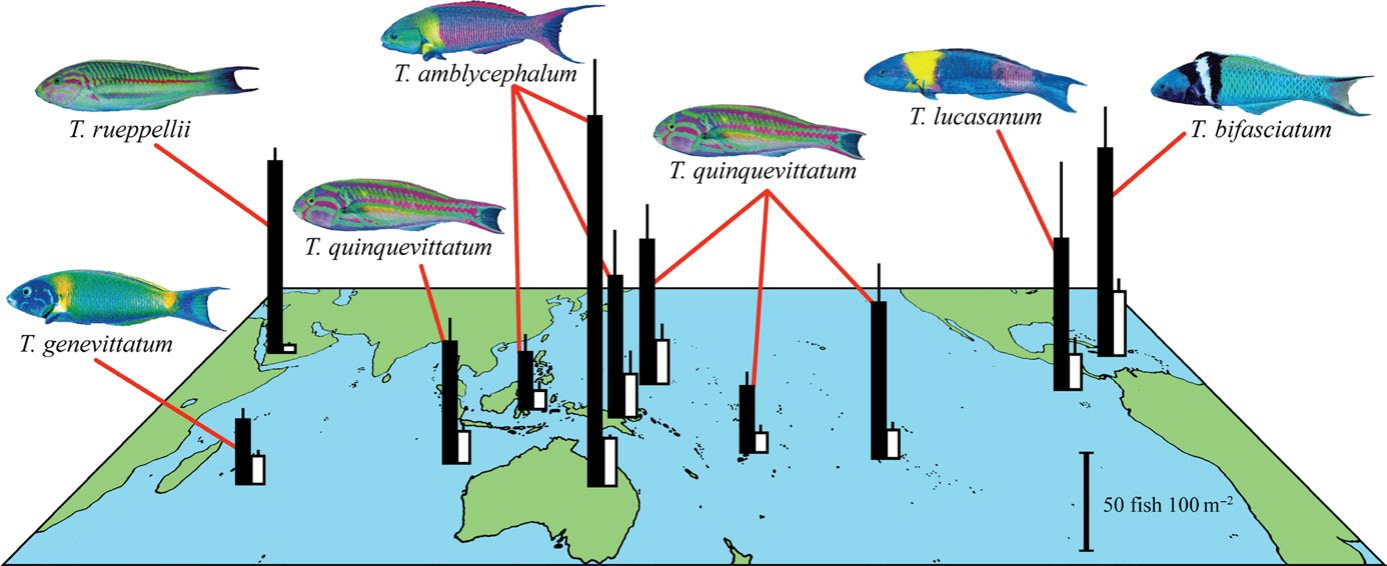
Mean density (± standard error) of *Thalassoma* (black bars) and fishes of all other labrid genera (white bars) occupying wave‐swept crest habitats at eleven coral reef locations (Table 1). Images are of the dominant species (all were *Thalassoma*) at each location

**Figure 2:**
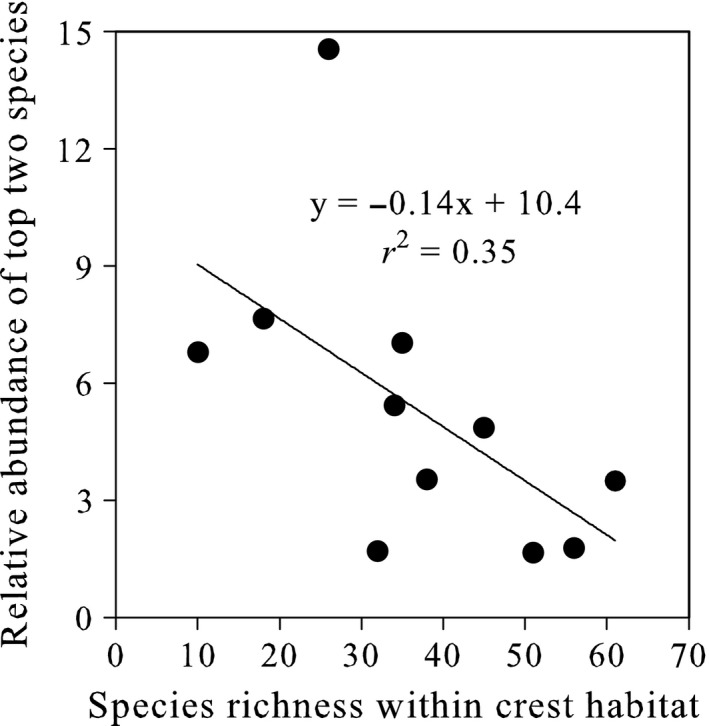
Numerical dominance of *Thalassoma* fishes across a global gradient in labrid species richness within wave‐swept crest habitats, as indicated by their relative abundance (mean density of the most abundant species, which was always a *Thalassoma*, divided by mean density of the next most abundant species, Table 1) across eleven coral reef locations

Shallow wave‐swept coral reef habitats around the world were overwhelmingly dominated by fishes of the genus *Thalassoma* (Figure 1). Collectively, *Thalassoma* fishes comprised 63 ± 4% (mean ± SE, *n* = 11 locations) of all labrid individuals within exposed coral reef crests (Figure 1), where *Thalassoma* was up to 15 times more abundant than any other labrid. The most abundant species at each of our locations was always a *Thalassoma*, which on average (across all locations, ± SE) had a density that was 5.3 ± 1.1 times higher than the next most abundant labrid species. The extent of numerical dominance by the top species increased along a gradient in species richness, with the greatest dominance evident in low‐diversity systems (Figure 2). The dominant *Thalassoma* species in the east Pacific and Atlantic (with 10–18 reef crest species) was seven to eight times more abundant than their confamiliars, versus two to three times more abundant in Indonesia and Papua New Guinea (with 56–61 species; Figure 2, Table 1). The dominant species at each location possessed very high pectoral fin aspect ratios at the upper extreme (2.08) for this family (Table 1).

**Figure 3:**
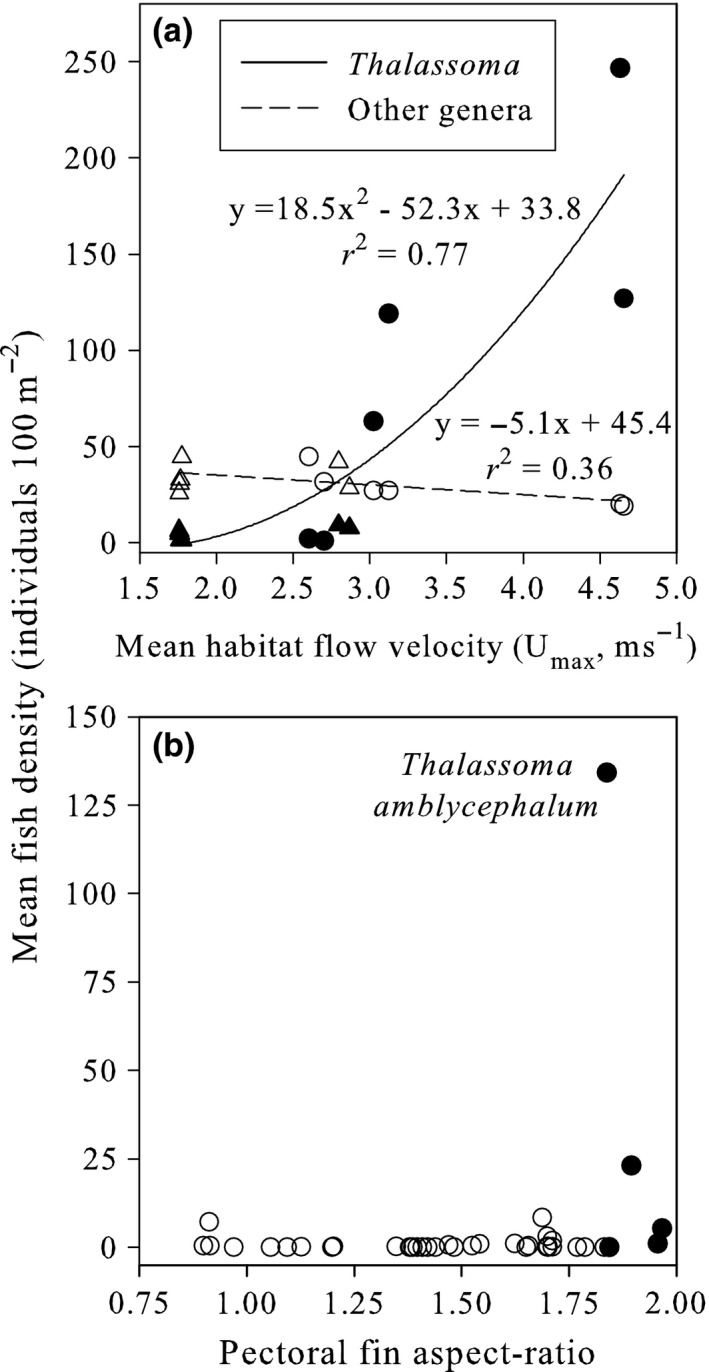
(a) Divergent relationships between mean water flow speed and fish density for *Thalassoma* (filled symbols) and fishes of all other labrid genera (open symbols) occupying shallow coral reef habitats that are either sheltered (back reef = triangles) or exposed (crest = circles) to wave energy across six reefs spanning a 40‐km cross‐shelf cline on the northern Great Barrier Reef. (b) Within reef crests on the outer Great Barrier Reef that are fully exposed to oceanic wave energy, one species of *Thalassoma* (all species of this genus are denoted by filled circles) with a high pectoral fin aspect ratio had a density more than five times higher than the next most abundant species (another *Thalassoma*), and over ten times higher than any other labrid genera (open circles) in this habitat

In exploring these patterns across a geographical cline in wave energy, we found *Thalassoma* only dominated the most exposed mid‐ and outer‐shelf reef crest habitats, which experienced high habitat flow velocities above 3 m s^−1^ (Figure 3a). Grossly outnumbered by other labrids within the sheltered inshore and back reef habitats, *Thalassoma* densities increased nonlinearly with habitat flow velocity, while other labrids decreased in abundance (Figure 3a), to produce a significant habitat by shelf interaction (Table 2). In the most exposed wave‐swept reef crest habitats on the outer Great Barrier Reef, the labrid assemblage was dominated by the single species *Thalassoma amblycephalum*, which had one of the highest pectoral fin aspect ratios for this assemblage (Figure 3b). The density of *T. amblycephalum* was more than five times higher than the next most abundant species (another *Thalassoma*), and over ten times more abundant than species of all the other labrid genera (Figure 3b).

**Table 2:** Summary of PERMANOVA comparing relative abundance of *Thalassoma* and other labrid fishes among reefs, habitat zones, and shelf positions of varying wave exposure on the Great Barrier Reef

Source	*df*	SS	MS	Pseudo‐*F*	*p*‐value
Shelf	2	14.33	7.16	1,964.00	<.01
Habitat	1	3.15	3.15	154.60	<.01
Reef (Shelf)	3	0.01	0.004	0.46	.36
Shelf*Habitat	2	0.39	0.20	9.68	.04
Reef(Shelf)*Habitat	3	0.06	0.02	2.59	.27
Residual	36	0.28	0.008		
Total	47	18.23			

## 4 Discussion

Fishes from the genus *Thalassoma* have achieved remarkable ecological success in wave‐swept habitats around the globe. In a pattern repeated by multiple species on coral reefs spanning three major ocean basins and the Red Sea, *Thalassoma* have attained abundances at least three and up to 15 times greater than all other labrid species occupying exposed shallow reef habitats. Extreme levels of wave‐driven water motion appear crucial to this ecological success, with *Thalassoma* dominance only occurring within the most wave‐swept habitats of one of the most diverse regions in our study, the Great Barrier Reef. Whether through ecological opportunity or release, *Thalassoma* seem to have relaxed the abiotic and biotic selection pressures acting on other labrids attempting to exploit the rich resources available in these challenging habitats.

Winged (high aspect ratio) pectoral fins have emerged as a significant adaptation in the biology and ecology of several independent reef fish lineages (Fulton et al., 2005; Wainwright et al., 2002) and may be a key factor in the ecological release of *Thalassoma* on wave‐swept reefs. Earlier suggestions that high aspect ratio fins are related to incident wave energy (Bellwood & Wainwright, 2001; Fulton et al., 2005) are supported, and our quantitative observations reveal a nonlinear relationship of increasing dominance of *Thalassoma* in wave‐swept crest habitats according to the magnitude of wave‐driven flows. By flapping their tapered pectoral fins to achieve a fast and efficient form of underwater flight, *Thalassoma* fishes are able to meet the challenges of this demanding hydrodynamic environment, while minimizing the costs of locomotion over a broad range of swimming speeds (Fulton, 2010; Fulton et al., 2013a; Wainwright et al., 2002). Such energy savings (compared to related species without tapered pectoral fins) could be considerable, as swimming activity can be a major component of the daily energy and activity budgets of fishes (e.g., Boisclair & Sirois, 1993; Layton & Fulton, 2014). When foraging in wave‐swept habitats rich with resources (e.g., crustacean prey, Kramer et al., 2014), this efficient high‐speed locomotor system could be pivotal for *Thalassoma* to achieve high population sizes.

Competitive pressure among congeners could be influencing the relative extent of ecological release and population sizes attained by *Thalassoma* in wave‐swept habitats. In locations such as Panama and the Red Sea, we found a single *Thalassoma* species was dominant at densities seven to fifteen 15 times higher than the next most abundant species. However, in locations of higher species richness, such as the Great Barrier Reef and Papua New Guinea, the relative dominance of the top *Thalassoma* species was lower (two to five times the next species), possibly because the next most abundant species was often another *Thalassoma*. Presumably, this pattern is influenced by the tendency for close relatives to be most ecologically similar and compete most strongly for the same resources (Cahill et al., 2008; Darwin, 1859; Webb et al., 2002). *Thalassoma* tend to occupy and access similar microhabitats and food resources within shallow reef habitats (Fulton, Bellwood, & Wainwright, 2001; Fulton & Bellwood, 2002; Bellwood, Wainwright, Fulton, & Hoey, 2006). Notably, in locations where we found more than one *Thalassoma* species in abundance, the numerically dominant species was often *T. quinquevittatum*, which has the highest fin aspect ratio of the genus (Bellwood & Wainwright, 2001; Wainwright et al., 2002). Possession of a higher aspect ratio pectoral fin than their congeners may have conferred enough competitive advantage for *T. quinquevittatum* to dominate within wave‐swept habitats (cf. Schoener, 1974), particularly in oceanic locations that are likely to experience the most extreme levels of daily wave action. One notable exception is *T. amblycephalum*, which exhibits numerical dominance on the reef crests of Indonesia, Papua New Guinea and the Great Barrier Reef, despite having a slightly lower fin aspect ratio than the congenerics at these locations. Notably, these locations in the central Indo‐Australian Archipelago can be considered lower in relative wave exposure than the other open‐ocean locations we sampled across the Indo‐Pacific (Gove et al., 2013; Hemer, Katzfey, & Trenham, 2013). Lower relative wave exposure could relax the intensity of selection upon fin shape as a key trait for dominance, whereby other traits such as trophic resource use may help explain patterns of species dominance at the global scale.

Trophic versatility may be one such trait that helps explain the global success of *Thalassoma*, especially considering the other genera that possess winged pectoral fins but have not achieved such numerical dominance. With few exceptions (*T. amblycephalum* specialized toward the abundant resource of planktonic crustaceans), *Thalassoma* fishes are known for their flexible trophic morphology and broad dietary preferences relative to their confamiliars, and generalized foraging among a wide spectrum of coral reef microhabitats (Bellwood et al. 2006, Fulton & Bellwood, 2002; Berkström, Jones, McCormick, & Srinivasan, 2012). Such versatility should facilitate prey‐switching among the most common resource types within a given habitat (Murdoch, 1969; Kondoh, 2003; Bellwood et al. 2006), which would help *Thalassoma* to rapidly exploit trophic resources, and ultimately, outnumber sister taxa with similarly winged pectoral fins but more select trophic needs (e.g., *Gomphosus—*brachyuran crabs within coral heads, *Stethojulis*—microcrustaceans within sediment beds; Fulton & Bellwood, 2002). Notably, another *Thalassoma* species (*T. lunare*) with broad trophic preferences, but rounded pectoral fins, is one of the most common labrids within wave‐sheltered and deeper reef habitats across tropical and temperate latitudes (Fulton & Bellwood 2004, Bernardi, Bucciarelli, Costagliola, Robertson, & Heiser, 2004, Berkström et al., 2012). Collectively, it appears *Thalassoma* exhibit a range of traits that predispose them to success within a range of habitat types to which they gain access according to their locomotor capabilities.

Colonization of wave‐swept habitats by fishes appears to have been a relatively recent phenomenon in the evolution of coral reef ecosystems, with an initial invasion in the early Miocene about 23–20 Ma (Bellwood, Goatley, & Bellwood, 2016). *Thalassoma* may have been a key component of this invasion, which coincided with a wave of phenotypic innovations among the Labridae, and speciation of *Thalassoma* (Bernardi et al., 2004; Cowman, Bellwood, & van Herwerden, 2009). Exceptional swimming capabilities appear to have combined with trophic versatility to allow *Thalassoma* to gain a competitive edge in exploiting challenging high‐flow environments around the world. For *Thalassoma*, it appears the energy savings afforded by a highly tapered pectoral fin have allowed the relaxation of environmental selection and an ecological release in shallow wave‐swept habitats, where the ecological success of *Thalassoma* has manifested in the global domination of these extreme coral reef habitats.

## Conflict of Interest

We have no conflict of interests to declare.

## Data accessibility

Supporting data is available in Table 1 and via Dryad doi:10.5061/dryad.24q8p.
